# Magnesium and Morphine in the Treatment of Chronic Neuropathic Pain–A Biomedical Mechanism of Action

**DOI:** 10.3390/ijms222413599

**Published:** 2021-12-18

**Authors:** Kamila Kulik, Barbara Żyżyńska-Granica, Agnieszka Kowalczyk, Przemysław Kurowski, Małgorzata Gajewska, Magdalena Bujalska-Zadrożny

**Affiliations:** 1 Centre for Preclinical Research and Technology, Department of Pharmacodynamics, Medical University of Warsaw, Banacha 1b Str., 02-097 Warsaw, Poland; bzyzynska@wum.edu.pl (B.Ż.-G.); akowalczyk@wum.edu.pl (A.K.); przemyslaw.kurowski@wum.edu.pl (P.K.); magdalena.bujalska@wum.edu.pl (M.B.-Z.); 2 Chair and Department of Biochemistry, Medical University of Warsaw, Banacha 1 Str., 02-097 Warsaw, Poland; 3 Department of Physiological Sciences, Warsaw University of Life Sciences, Nowoursynowska 159 Str., 02-776 Warsaw, Poland; malgorzata_gajewska@sggw.edu.pl

**Keywords:** magnesium, morphine, N-methyl-D-aspartate receptor, µ-opioid receptor, receptor association, analgesia, neuropathic rats

## Abstract

The effectiveness of opioids in the treatment of neuropathic pain is limited. It was demonstrated that magnesium ions (Mg^2+^), physiological antagonists of N-methyl-D-aspartate receptor (NMDAR), increase opioid analgesia in chronic pain. Our study aimed to determine the molecular mechanism of this action. Early data indicate the cross-regulation of µ opioid receptor (MOR) and NMDAR in pain control. Morphine acting on MOR stimulates protein kinase C (PKC), while induction of NMDAR recruits protein kinase A (PKA), leading to a disruption of the MOR-NMDAR complex and promoting functional changes in receptors. The mechanical Randall-Selitto test was used to assess the effect of chronic Mg^2+^ and morphine cotreatment on streptozotocin-induced hyperalgesia in Wistar rats. The level of phosphorylated NMDAR NR1 subunit (pNR1) and phosphorylated MOR (pMOR) in the periaqueductal gray matter was determined with the Western blot method. The activity of PKA and PKC was examined by standard enzyme immunoassays. The experiments showed a reduction in hyperalgesia after coadministration of morphine (5 mg/kg intraperitoneally) and Mg^2+^ (40 mg/kg intraperitoneally). Mg^2+^ administered alone significantly decreased the level of pNR1, pMOR, and activity of both tested kinases. The results suggest that blocking NMDAR signaling by Mg^2+^ restores the MOR-NMDAR complex and thus enables morphine analgesia in neuropathic rats.

## 1. Introduction

Despite the fact that the last few decades have brought progress in the treatment of many diseases, the relief of neuropathic pain is still one of the major challenges in medicine. The barrier in the alleviation of this type of pain is the limited efficacy of classic analgesics, including opioids [1,2,3]. A reduction in opioid analgesia appears to be associated with the increased activation of pronociceptive N-methyl-D-aspartate receptors (NMDARs) during the development of neuropathic pain [4,5].

One of the solutions is the administration of opioids in high doses [6], which unfortunately increases the risk of side effects, such as respiratory depression, opioid hyperalgesia, constipation, rapid development of tolerance, and spreading in recent years addiction risk [7,8,9,10].

Data available in the literature indicate that synthetic NMDAR antagonists not only alleviate neuropathic pain [11,12,13,14,15] but also enhance the analgesic effect of opioids [16,17,18]. It is worth noting that magnesium ions (Mg^2+^) are the physiological antagonists of the NMDAR channel, and their analgesic properties have been demonstrated both in preclinical [11,19,20] and clinical [21,22] studies in neuropathic pain. Mg^2+^ have relatively mild side effects at therapeutic doses and are considered safer and better-tolerated compounds than synthetic NMDAR antagonists. Importantly, several animal studies have reported that Mg^2+^ increase opioid analgesia in chronic neuropathic pain [23,24,25,26]. However, the cellular mechanism of this interaction has not been elucidated yet.

Considering the fact that µ-opioid receptor (MOR) is the primary and most widely studied mediator of opioid activity, including morphine (MRF), our investigations focus on this receptor. MOR belongs to the group of metabotropic G protein-coupled receptors (GPCRs). The signaling efficiency of this receptor is modulated and finally limited by phosphorylation of the appropriate intracellular amino acid residues. It was established that MOR phosphorylation precedes a process of receptor desensitization, uncoupling, and internalization [27]. It is noteworthy that MOR can undergo heterologous agonist-independent as well as homologous agonist-induced phosphorylation. Two protein families may phosphorylate the MOR residues: G protein-coupled receptor kinases (GRKs), as well as second messenger-dependent protein kinases including protein kinase C (PKC) and protein kinase A (PKA). It has been shown that serine 375 (Ser375) is the initiating residue in a hierarchical phosphorylation cascade [28,29]. In turn, NMDARs are ionotropic glutamate receptors, which form tetrameric complexes typically containing two essential NR1 subunits assembling with two modulatory NR2 subunits [30,31]. The function of NMDAR, including channel properties and localization at synapses, is also regulated by protein phosphorylation. It has been identified that NR1 C-terminus is phosphorylated via PKC on two serine residues (Ser890 and Ser896), while a neighboring site Ser897 is phosphorylated by PKA [32]. The C-terminus domains of NMDAR NR2 subunits (especially NR2A/B) are large, and they contain multiple amino acid sites that are phosphorylated by PKA, PKC, cyclin-dependent kinase-5 (Cdk5), calcium calmodulin-dependent kinase (CaMKII), casein kinase (CK2), Src and/or Fyn non-receptor tyrosine kinases [33]. What is important, studies have shown that activation of PKA and PKC potentiates the amplitude of NMDAR-mediated currents [34,35].

During recent years different groups have convincingly demonstrated the functional cross-regulation of MORs and NMDARs in pain control [36,37,38,39,40]. It was shown that NMDAR and MOR colocalize on the cell membranes of some postsynaptic central nervous system (CNS) structures, with particular concentration in the periaqueductal gray matter (PAG). In the resting state, MOR is linked to the NR1 subunit of NMDAR via the C1 segment of the NR1 C-terminus [39]. Importantly, MOR or NMDAR NR1 phosphorylation leads to a dissociation of these two proteins and NMDAR activation. In detail, MRF through MOR-Gβ/γ-PI3K-Akt-nNOS signaling pathway stimulates the production of NO [41]. Increased concentration of NO activates endogenous reserves of zinc ions, which are necessary for the recruitment of PKC [42,43]. Then, PKC phosphorylates serine residues in the C1 segment, separates the MOR-NMDAR complex, and produces the sustained potentiation of NMDAR calcium (Ca^2+^) currents [39]. Ca^2+^ ions influx through the NMDAR-activated ion channel stimulates CaMKII and PKA [39,44]. PKA stimulation, as well as NMDAR and MOR separation, can occur both indirectly through PKC activation and directly in response to NMDAR agonist binding. The activated PKA promotes MOR serine residues phosphorylation and uncoupling of G-proteins from MOR. As previously mentioned, Ser375 is phosphorylated first and, in some cases, exclusively [45]. MOR or NMDAR stimulation (when they are co-localized) disrupts the MOR-NMDAR complex and impair MOR reactivity, reducing the analgesic efficacy of MRF [39]. Importantly, in the case of neuropathic pain, the increased expression of NMDAR is observed, and MOR-NMDAR association is reduced.

Interactions between MOR and NMDAR described above represent a highly promising basis for the development of more effective pharmacotherapy of pain. Garzon et al. extensively investigated the molecular mechanism of opioid tolerance and dependence development [46]. Since NMDARs are involved during neuropathic pain and opioids are less efficacious in this kind of pain, it was interesting to evaluate the influence of Mg^2+^, as NMDAR physiological antagonists, on MOR activity. The level of activation and inactivation of MOR and NMDAR after the tested compounds administration was investigated by analyzing phosphorylation of MOR Ser375 and NMDAR NR1 subunit Ser896 using the Western blot method. The activity of PKA and PKC involved in the bidirectional interaction between tested receptors was determined using standard enzyme immunoassays.

## 2. Results

### 2.1. The Influence of Mg^2+^ on the Analgesic Effect of Morphine in Streptozotocin-Induced Hyperalgesia after Mechanical Stimulation

**Figure 1:**
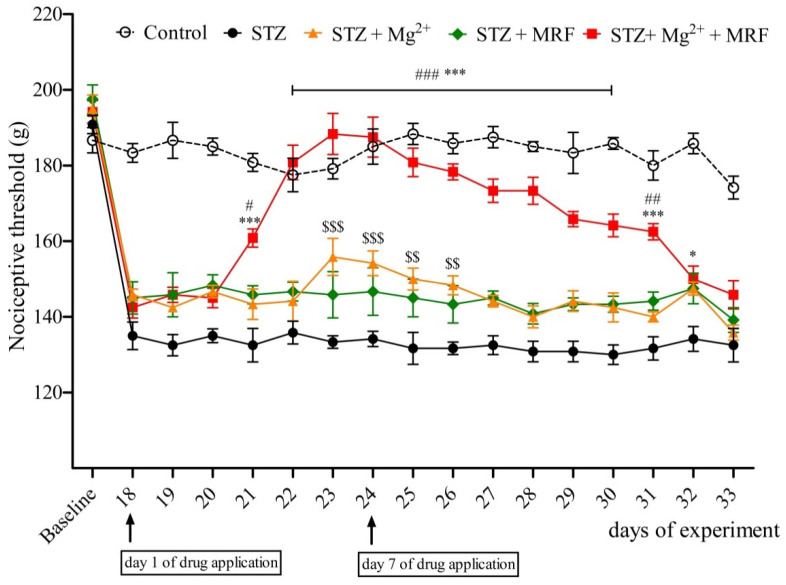
Influence of magnesium (Mg^2+^) on the analgesic activity of morphine (MRF) in streptozotocin (STZ)-treated rats. Days 18–24—drugs administration; days 19–25—measurements of prolonged activity of investigated drugs; days 26–33—measurements of pain threshold after discontinuation of drugs administration. Nociceptive thresholds, expressed in grams, were measured before STZ administration (baseline) and then before tested substances administration in rats with STZ-induced diabetic neuropathic pain from day 19 until day 25. Data are presented as means ± SEM. Statistical analysis was performed by two-way ANOVA followed by Bonferroni’s post-hoc test. *** *p* < 0.001, * *p* < 0.05 (STZ + Mg^2+^ + MRF vs. STZ), ### *p* < 0.001, ## *p* < 0.01, # *p* < 0.05 (STZ + Mg^2+^ + MRF vs. STZ + MRF), $$$ *p* < 0.001, $$ *p* < 0.01 (STZ + Mg^2+^ vs. STZ); *n* = 6 rats for each group.

**Figure 2:**
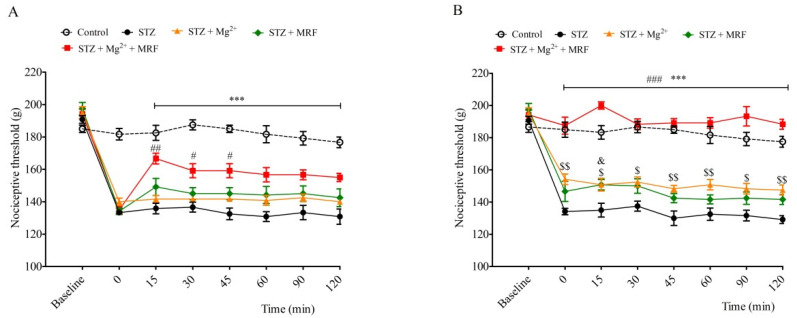
Effect of magnesium (Mg^2+^) on the antinociceptive activity of morphine (MRF) on the 1st day (Panel **A**) and the 7th day (Panel **B**) of the drugs’ application in streptozotocin (STZ)-induced diabetic neuropathic pain. Nociceptive thresholds, expressed in grams, were measured before STZ administration (baseline) and then before (0) and after tested substances administration in rats with STZ-induced diabetic neuropathic pain for 2 h. Data are presented as means ± SEM. Statistical analysis was performed by two-way ANOVA followed by Bonferroni’s post-hoc test. *** *p* < 0.001 (STZ + Mg^2+^ + MRF vs. STZ), ### *p* < 0.001, ## *p* < 0.01, # *p* < 0.05 (STZ + Mg^2+^ + MRF vs. STZ + MRF), $$ *p* < 0.01, $ *p* < 0.05 (STZ + Mg^2+^ vs. STZ), & *p* < 0.05 (STZ + MRF vs. STZ); *n* = 6 rats for each group.

Streptozotocin (STZ)-treated rats developed mechanical hyperalgesia within 2 weeks after single intramuscular administration of STZ. In turn, we recorded no changes in the nociceptive threshold in the control (healthy animals) group (data not shown). Mg^2+^ and MRF were administered daily for seven days from day 18 to day 24. Mg^2+^ applied alone to neuropathic animals caused an increase in the nociceptive threshold observed from day 23 until day 26 of the experiment (Figure 1). On the 18th day of the experiment (the 1st day of application), Mg^2+^ alone did not modify STZ-induced hyperalgesia (Figure 2A). However, on day 24 of the experiment (the 7th day of application), Mg^2+^ increased the nociceptive threshold over a period of 120 min after administration (Figure 2B). In turn, MRF administered alone on seven consecutive days did not change STZ-induced hyperalgesia (Figure 1). Similarly, no significant changes in nociceptive thresholds were observed on the 1st day of the drug’s application (day 18 of the experiment) over a period of 120 min after MRF administration (Figure 2A). However, on the 7th day (day 24 of the experiment), MRF alone induced a slight statistically significant analgesic effect 15 min after injection (*p* < 0.05) (Figure 2B). As it is shown in Figure 1, the mechanical hyperalgesia was significantly reduced in rats receiving the combination of Mg^2+^ and MRF starting from day 21 of the experiment (the third day of drugs application) compared to MRF-treated animals. After cessation of tested drugs (Mg^2+^ and MRF) application, animals’ pain sensitivity was reported to increase. On day 18 of the experiment (the 1st day of administration), simultaneous injection of Mg^2+^ and MRF increased the nociceptive threshold in comparison to rats receiving only MRF starting from 15 min after administration (166.66 ± 3.33 g and 149.167 ± 5.23 g for STZ + Mg^2+^ + MRF and STZ + MRF, respectively; Figure 2A). It is noteworthy that this effect was significantly higher on day 24 of the experiment (the 7th day of application) at the same time point (200 ± 2.23 g and 150.83 ± 3.27 for STZ + Mg^2+^ + MRF and STZ + MRF, respectively; Figure 2B).

### 2.2. Changes in the Expression of Phosphorylated Proteins Measured at the Serine Residues of N-methyl-D-aspartate Receptor NR1 Subunit and µ Opioid Receptor

**Figure 3:**
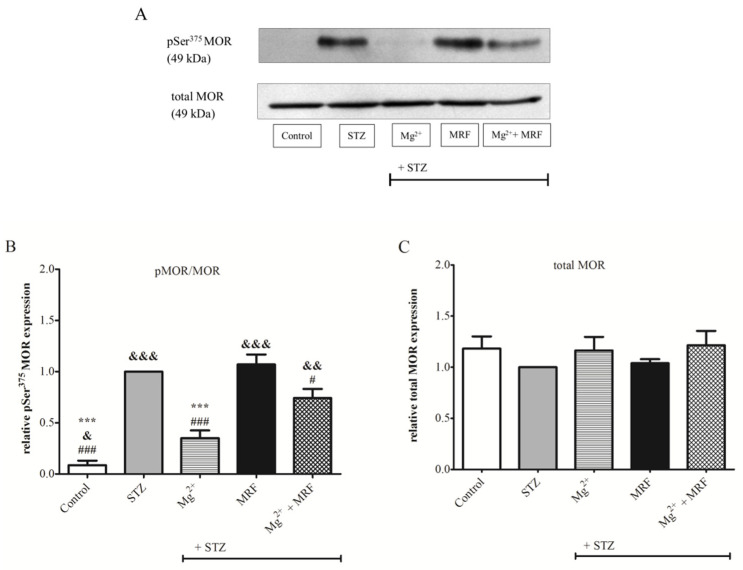
The effect of chronic administration of magnesium (Mg^2+^), morphine (MRF) and combination of these substances (Mg^2+^ + MRF) in periaqueductal gray matter (PAG) lysate from streptozotocin (STZ)-treated rats on the level of serine 375 (Ser375) µ opioid receptor (MOR) phosphorylation. (**A**) Representative result of the Western blot analysis showing phosphorylated serine 375 (pSer375) MOR (top panel) and total MOR (bottom panel). (**B**) The level of protein phosphorylation at the Ser375 residue expressed as the ratio of the phosphorylated form to the total form of the tested proteins (pMOR/MOR). (**C**) Changes in the expression of total MOR. Statistical analysis was performed using one-way ANOVA followed by Dunnett’s post-hoc test. *** *p* < 0.001 vs. STZ; &&& *p* < 0.001; && *p* < 0.01; & *p* < 0.05 vs. STZ + Mg^2+^; ### *p* < 0.001; # *p* < 0.05 vs. STZ + MRF (graph **B**). There were no statistically significant differences in total MOR expression (*p* > 0.05; graph **C**); *n* = 6 rats/group.

**Figure 4:**
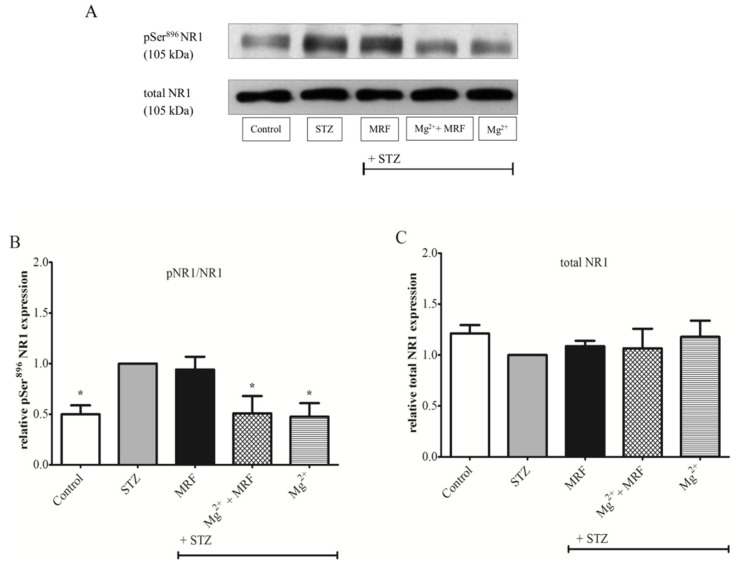
The effect of chronic administration of morphine (MRF), magnesium and morphine cotreatment (Mg^2+^ + MRF), and magnesium (Mg^2+^) in periaqueductal gray matter (PAG) lysate from streptozotocin (STZ)-treated rats on the expression of N-methyl-D-aspartate receptor (NMDAR) NR1 subunit phosphorylation. (**A**) Representative results of the Western blot assay showing phosphorylated serine 896 (pSer896) NR1 (top panel) and total NR1 (bottom panel). (**B**) The level of Ser896 phosphorylation expressed as the ratio of the phosphorylated form to the total form of the tested proteins (pNR1/NR1). (**C**) Changes in the expression of total NR1. Statistical analysis was performed using one-way ANOVA followed by Dunnett’s post-hoc test. * *p* < 0.05 vs. STZ (graph **B**). There were no statistically significant differences in total NR1 (*p* > 0.05; graph **C**); *n* = 6 rats/group.

To determine changes in the level of protein phosphorylation at the Ser375 MOR (Figure 3) and at the Ser896 NMDAR NR1 subunit (Figure 4), we used the Western blot technique in PAG lysate. As shown in Figure 3B, phosphorylation of Ser375 at MOR for STZ-treated rats was significantly higher compared to the control group (healthy animals). Importantly, Mg^2+^ administered alone in rats with STZ-induced diabetic neuropathic pain resulted in a statistically significant decrease in the protein phosphorylation on Ser375 MOR in relation to the STZ group (0.35 ± 0.08 vs. 1 for STZ group; *p* < 0.001). MRF injected alone showed no changes in the phosphorylation of tested protein compared with the STZ group (1.07 ± 0.1 vs. 1 for the STZ group; *p* > 0.05). In turn, simultaneous administration of Mg^2+^ and MRF caused a significant reduction in the phosphorylation of MOR compared to the STZ group receiving MRF alone (0.74 ± 0.09 vs. 1.07 ± 0.1; *p* < 0.05). In the case of ionotropic NMDAR (Figure 4B), high phosphorylation of the Ser896 NR1 subunit was observed in STZ-treated rats compared to the control group (*p* < 0.05). Treating STZ animals with MRF did not affect the phosphorylation of the NR1 in relation to the STZ group (0.94 ± 0.13 vs. 1, *p* > 0.05). In contrast, coadministration of Mg^2+^ and MRF generated a statistically significant decrease in the level of NR1 phosphorylation in rats with STZ-induced diabetic neuropathic pain in comparison to the non-treated STZ group (0.51 ± 0.17 vs. 1; *p* < 0.05). A similar effect was observed after Mg^2+^ administered alone (0.48 ± 0.13 vs. 1; *p* < 0.05). The expression of total MOR (Figure 3C) and total NR1 (Figure 4C) did not change in all tested groups.

### 2.3. Changes in the Activity of Protein Kinase A and Protein Kinase C in the Streptozotocin-Treated Rats after Administration of Tested Compounds

**Figure 5:**
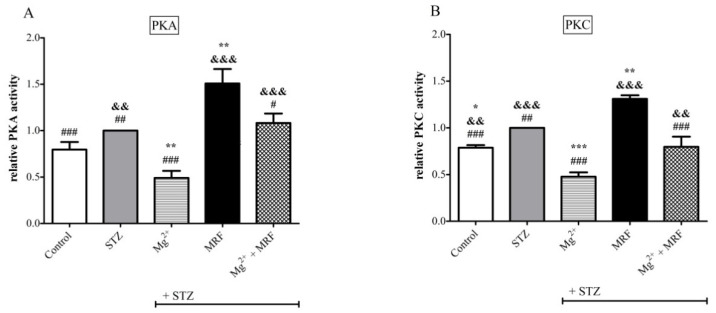
Changes in the level of (**A**) protein kinase A (PKA) and (**B**) protein kinase C (PKC) in response to magnesium (Mg^2+^), morphine (MRF) and the combination of these substances (Mg^2+^ + MRF) in periaqueductal gray matter (PAG) lysate from streptozotocin (STZ)-treated rats. Results are presented as means ± SEM. Statistical analysis was performed using one-way ANOVA followed by Dunnett’s post-hoc test. *** *p* < 0.001; ** *p* < 0.01; * *p* < 0.05 vs. STZ; ### *p* < 0.001; ## *p* <0.01, # *p* < 0.05; vs. STZ + MRF; &&& *p* < 0.001, && *p* < 0.01 vs. STZ + Mg^2+^; *n* = 6 rats/group.

As shown in Figure 5B, an increase in the activity of PKC was observed in STZ-treated rats compared to the control animals. In turn, no statistically significant changes were recorded in levels of PKA in these two groups (Figure 5A). However, for both PKA and PKC, the 7-day application of Mg^2+^ led to a reduction in their activities (0.49 ± 0.08 vs. 1, *p* < 0.01 for PKA and 0.48 ± 0.05 vs. 1, *p* < 0.001 for PKC). Importantly, the application of MRF to animals with chronic neuropathic pain resulted in an increase in the activity of both kinases compared to the STZ group (1.51 ± 0.16 vs. 1 for PKA and 1.31 ± 0.04 vs. 1 for PKC; *p* < 0.01). Simultaneous administration of Mg^2+^ and MRF showed no changes in the level of PKA and PKC (1.08 ± 0.1 for PKA and 0.8 ± 0.11 for PKC, *p* > 0.05) in relation to the STZ group. However, statistically significant changes in the activity of both enzymes were observed after injection of Mg^2+^ and MRF in relation to MRF alone (1.08 ± 0.1 vs. 1.51 ± 0.16 for PKA, *p* < 0.05 and 0.8 ± 0.11 vs. 1.31 ± 0.04 for PKC, *p* < 0.001).

## 3. Discussion

Despite medical advances of the last few decades, opioids are still one of the most powerful groups of analgesics. These compounds are used not only in the treatment of cancer-related pain and acute postoperative pain but also, with uncertain effectiveness, in the treatment of neuropathic pain [47]. In clinical practice, a regimen known as co-analgesia is employed to increase the analgesic efficiency of opioids as well as to reduce their doses and thereby avoiding the risk of side effects. NMDAR antagonists are widely studied co-analgesics, and coadministration of Mg^2+^ with opioids has received particular interest. However, despite many reports regarding the beneficial interaction between Mg^2+^ and opioids [48], the mechanism of this phenomenon still remains to be elucidated.

In our studies, we focused on PAG structure, which is a part of the descending pain modulatory pathway and is involved in the opioid control of nociception [49]. Moreover, since MOR and NMDAR colocalize on the cell membranes in PAG, but in neuropathic pain, NMDAR activity negatively affects the signaling capacity of MOR, we decided to investigate the activities of both mentioned receptors after administration of Mg^2+^ and MRF.

The starting point for characterization of this interaction was a behavioral study investigating the analgesic potential of coadministration of tested drugs in the model of STZ-induced diabetic neuropathic pain. We studied the antihyperalgesic effect of tested drugs after the chronic administration as well as an analgesic response over a period of 120 min directly after the drugs’ application on day 18 and day 24 of the experiment using mechanical stimulus. Some data indicate that PAG stimulation was effective in mechanical allodynia in the animal model of neuropathic pain [50]. As shown in our previous studies [25,51], the gradual development of mechanical hyperalgesia was observed in STZ-treated animals (data not shown). We demonstrated a lack of antinociceptive effect upon MRF application alone over 7 consecutive days in animals with persistent neuropathic pain. Moreover, the nociceptive threshold data measured over a period of 120 min after MRF administration on the 1st day of application did not modify STZ-induced hyperalgesia (Figure 2A). However, on day 7 of consecutive MRF administration, the antinociceptive activity of the opioid occurred 15 min after the injection (Figure 2B). The weak analgesic activity of MRF has also been observed in our previous studies in different kinds of neuropathic pain [24,25]. Moreover, other authors have shown that MRF administered alone induced no change in the nociceptive threshold in diabetic rats or fairly modest antihyperalgesic effect in mononeuropathic animals [23]. In contrast, Mg^2+^ applied alone elevated the mechanical pain threshold in the group of animals with diabetic hyperalgesia (Figure 1). This is probably the result of chronic use of subtherapeutic doses of Mg^2+^, which gradually inhibit the NMDAR hyperactivity. Additionally, on day 7 of application, this analgesic effect lasted over 2 h after Mg^2+^ administration (Figure 2B). The results of these studies are not surprising since similar effectiveness of Mg^2+^, as well as synthetic NMDAR antagonists, has been demonstrated by other laboratories in neuropathic pain [11,19,23,52]. Importantly, it was also demonstrated that coadministration of Mg^2+^ with MRF produced a significant reduction in mechanical hyperalgesia in STZ-treated rats after systemic few days of administration. This effect was also maintained for several days after cessation of drug administration. Similar results have been observed in previous studies in a vincristine neuropathic model of pain [24]. This is potentially associated with the action of morphine-6glucuronide (M6G), the analgesic active metabolite of MRF, which affects MOR [53]. M6G, during long-term MRF administration, may reach sufficiently high concentrations in CNS and cause longer-lasting pain relief compared to MRF [53,54]. We can speculate that Mg^2+^ blocking of the NMDAR signaling and the likely unblocking of MOR activity allows for the prolonged action of MRF metabolites. Comparing Mg^2+^ cotreatment with MRF to the inferior analgesic effect observed after separate administration of both compounds, the coadministration of these substances appears to be highly effective in the alleviation of neuropathic pain. Results of this study confirm previous observations on the efficacy of such combination in relieving various types of neuropathic pain [23,24,25,26]. Thus, the intensification of opioid analgesia by Mg^2+^ can provide an opioid-sparing dose, which in turn allows for the reduction in side effects. Unfortunately, as we mentioned above, the nature of synergistic analgesic interactions between opioids and Mg^2+^ remains unclear.

One of the most important processes regulating the receptor’s activity is the covalent modification of receptors by their phosphorylation or dephosphorylation [27]. In the case of the examined receptors, their phosphorylation results in metabotropic MOR inactivation and ionotropic NMDAR stimulation. Therefore, in our studies, the activity of the above-mentioned receptors was determined by evaluation changes in protein phosphorylation. It was observed that the phosphorylation of both, Ser375 at the C-terminus MOR (Figure 3B) and Ser896 of the NMDAR NR1 subunit (Figure 4B), was significantly higher in STZ-treated rats than in control animals. Our results are in agreement with those of Rondon et al., who also observed NMDARs hyperactivity based on increased Ser896 phosphorylation in STZ-treated rats [19]. The high levels of an inactive, phosphorylated form of MOR detected in STZ-treated rats explain the lack of effectiveness of MOR agonists, such as MRF, in treating neuropathic pain. In addition, the phosphorylation of Ser375 at MOR after chronic administration of MRF to STZ-treated animals was comparable to the diabetic rats. These results are in line with our behavioral data showing poor analgesic properties of MRF in the neuropathic model of pain.

Importantly, our data showed that chronic administration of Mg^2+^ in rats with STZ-induced diabetic neuropathic pain significantly reduced the level of Ser375 phosphorylation at MOR in relation to STZ-treated animals (Figure 3B). Moreover, we demonstrated significantly reduced NMDAR phosphorylation after chronic application of Mg^2+^ in STZ-treated rats to levels observed in control animals (Figure 4B). These results, together with behavioral observations, indicate that blocking NMDAR signaling by Mg^2+^ induces the MOR-NMDAR re-association and restores the analgesic efficacy of MRF in neuropathic pain. Furthermore, our data revealed that the simultaneous administration of MRF and Mg^2+^ caused a reduction in Ser375 phosphorylation in comparison to rats receiving MRF alone. However, the Ser375 phosphorylation was greater than in the group treated only with Mg^2+^. The data presented above demonstrate the concurrent activity of the signaling pathways involving both receptors. MOR indirectly dephosphorylated due to the activity of Mg^2+^ can bind MRF and transmit the signal. Subsequently, after signal transmission, the phosphorylation of MOR is again elevated. Thus, this observation seems to explain the mechanism of unblocking morphine’s efficacy by Mg^2+^.

In our studies, we also examined the activity of PKA and PKC involved in the bidirectional regulation between MOR and NMDAR. Surprisingly, we found that chronic administration of MRF to STZ-treated rats significantly increased the activity of both kinases compared to untreated diabetic animals, although the level of MOR phosphorylation suggested its inactivation in a state of neuropathic pain. Nevertheless, we note that MRF is a nonselective agonist, and it influences the activity of kinases through other receptor subtypes. Moreover, it was established that MORs and δ opioid receptors (DORs) colocalize in the same axonal terminals of some CNS structures, including PAG and spinal cord [55,56,57]. A number of studies have shown that MOR and DOR undergo cross-regulation, and the DOR has a negative influence on MOR function [58]. Particularly, the administration of DOR antagonists decreases the development of MRF-induced tolerance and dependence [59]. According to Garzon et al., MRF preferentially influences MOR, but DOR can also be involved in the chronic responses to MRF [46]. Moreover, data described by Gucker and Bidlack [60] showed that PKC can play a significant role in DOR activity. They revealed that MRF in the presence of a PKC activator increases DOR down-regulation in vitro. Additionally, in our behavioral studies, a slight analgesic effect was observed 15 min after injection on day 7 of the chronic MRF administration. Thus, we suspect that in our studies increase in PKC and, consequently, PKA activity compared to the STZ group could be partially associated with the activation of DOR.

Importantly, our study revealed that chronic administration of Mg^2+^ causes a significant reduction in the activities of PKA and PKC in STZ-treated animals in relation to diabetic rats (Figure 5). It could be speculated that NMDAR inhibition promoted the reduction in PKA level and consequently decreased PKC activity. Data available in the literature indicate that activation of PKC and PKA may cause NMDAR NR1 Ser896 and Ser897 residues phosphorylation [61,62], further supporting the evidence that intracellular mechanisms activate NMDAR. Moreover, simultaneous induction of both kinases results in the transfer of newly synthesized NMDAR subunits from the cytosolic fraction to the membrane leading to central sensitization [63]. Thus, reduced activity of both enzymes after chronic Mg^2+^ stimulation may inhibit the transfer of NMDAR subunits from the cell interior to the cell membrane.

In the group receiving the combination of both tested drugs, the activity of PKA and PKC was statistically significantly lower than after MRF administration alone but significantly higher compared to diabetic rats treated only with Mg^2+^. Thus, it can be concluded that the NMDAR inhibition by Mg^2+^ and consequently the reduction in PKA level restores the activity of MOR. In addition, PKA-induced heterologous MOR phosphorylation is reduced by Mg^2+^, which corresponds to reduced PKC activity observed in our study. The combination of Mg^2+^ and MRF enables MRF to stimulate MOR, and consequently, a greater PKC activation was observed, representing the beginning of the cascade leading to heterologous MOR phosphorylation and its inactivation.

Interestingly, in our studies, we used the systemic route of drug administration and demonstrated the antinociceptive effect of the simultaneous use of Mg^2+^ and MRF. As both of investigated drugs cross the blood-brain barrier [64,65] and MOR and NMDAR colocalize in PAG, we suggest that the mechanism of this interaction is direct in PAG neurons.

## 4. Materials and Methods

### 4.1. Ethical Approval

All animal procedures were conducted in accordance with the guidelines published in the European directive 2010/63/EU on the protection of animals used for scientific purposes. The protocols were approved by the II Ethical Committee for Experiments on Small Laboratory Animals, Medical University of Warsaw (permit numbers: 34/2012 and 22/2015), as well as the I Ethical Committee for Experiments on Animals, University of Warsaw (permit number: 497/2017).

### 4.2. Laboratory Animals

Experiments were carried out on male Wistar rats weighing 250 to 280 g. The animals were housed in rooms maintained at a temperature of 22 ± 2 °C and humidity of 55% ± 10%. The rooms were equipped with an efficient mechanical ventilation system (15–20 air changes/h) and a 12 h light/12 h dark cycle. The rodents had free access to food and water. However, in an animal model of streptozotocin (STZ)-induced diabetic neuropathic pain, food was removed 16 h before STZ administration. Experimental groups consisted of at least 6 animals.

### 4.3. Drug Administration

Diabetes was induced by a single intramuscular administration of STZ (Cayman Chemical, Ann Arbor, MI, USA) at a dose of 40 mg/kg of body weight according to the method described previously [25,66]. Morphine sulfate (Polfa, Warsaw, Poland) and magnesium sulfate (MgSO_4_) (Polfa, Warsaw, Poland) were dissolved in 0.9% saline immediately before injection. Both tested compounds were applied intraperitoneally (i.p.). MRF was injected at a dose of 5 mg/kg, while MgSO_4_ at a dose of 40 mg/kg (i.e., 8 mg/kg Mg^2+^). The doses of MRF and MgSO_4_ were chosen based on our previous studies [25]. Control (healthy) rats were administered intraperitoneally with 0.9% saline.

### 4.4. Time Schedule

In behavioral experiments, all the tested drugs (except STZ administered only on day 1) were injected on 7 consecutive days (from day 18 to 24 after STZ was given) to rats with developed hyperalgesia. Mg^2+^ was applied daily 30 min before MRF injection. In experiments involving tissue harvest for Western blot analysis and enzyme-linked immunosorbent assays, the animals were killed by decapitation on day 24 of the experiment. Then, PAG was separated from the isolated brain and immediately immersed in liquid nitrogen.

### 4.5. Measurement of the Pain Threshold

**Figure 6:**
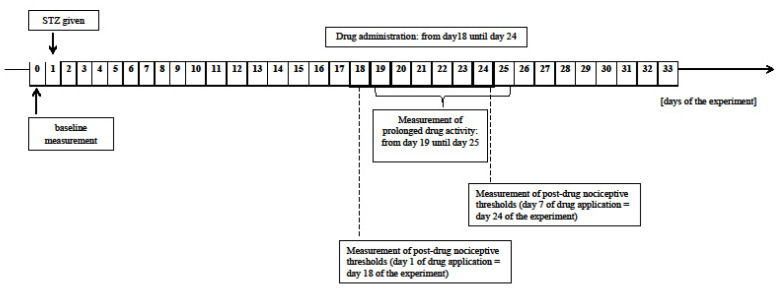
Scheme of the study design.

The changes in pain thresholds in the experimental animals after administration of tested substances were estimated using a mechanical stimulus, the Randall-Selitto paw withdrawal test as described previously [67]. The pain threshold obtained for each rat before STZ administration constituted the baseline. Nociceptive thresholds for the STZ-treated rats were measured each day after STZ administration (data not shown). Measurements of prolonged activity of the tested substances were carried out daily in rats with developed hyperalgesia for 7 consecutive days before drugs administration (from day 19 until day 25 of the experiment) and then after cessation of compounds application until the end of the experiment (day 33). The changes in nociceptive thresholds were also assessed at 15, 30, 45, 60, 90, and 120 min after the application of the investigated drugs (on the 1st and the 7th day of administration; day 18 and 24 of the experiment) (Figure 6).

Changes in pain thresholds after mechanical stimulation for each rat were expressed in grams as means ± SEM.

### 4.6. Preparation of Lysates for Western blot Analysis

The research material was prepared based on the method originally developed previously [68,69] with some modifications. All reagents were purchased from Sigma-Aldrich (St Louis, MO, USA) unless otherwise stated. PAG structures were homogenized on ice in 10 volumes of homogenization buffer (25 mM Tris-HCl (pH = 7.4), 1 mM EGTA, 0.32 M sucrose, 20 µM H-89 () as well as phosphatase inhibitor cocktail (P2850) and protease inhibitor mixture (P8340)). The homogenate was then centrifuged for 10 min at 1000× *g* to eliminate the nuclear fraction (pellet). The supernatant (S1) was centrifuged (20 min, 20,000× *g*) to obtain the second supernatant (S2) containing the cytoplasmic fraction and the pellet (P2). P2 was resuspended in 200 µL of homogenization buffer and centrifuged for 20 min at 20,000× *g*. The supernatant (S3) was discarded, and the pellet (P3), containing the membrane fraction, was resuspended in 25 mM Tris-HCl (pH 7.4) with protease inhibitors (0.2 mM PMSF, 2 µg/mL leupeptin, 0.5 µg/mL aprotinin). The P3 solutions containing 500 µg of protein were centrifuged (20 min, 20,000× *g*), and the obtained pellet was resuspended in 200 µL of an extraction buffer with the following composition: 50 mM Tris-HCl (pH 7.7), 50 mM NaCl, 1% Nonidet P-40, 20 µM H-89, phosphatase, and protease inhibitor cocktail. The samples were shaken for 12 h at 4 °C, centrifuged (10 min, 4 °C, 10,000× *g*) and the obtained supernatant was stored at −80 °C until further use. Before Western blot analysis, protein concentration was measured using the BCA method (Pierce™ BCA Protein Assay Kit, Thermo Fisher Scientific, Rockford, IL, USA).

### 4.7. Western blot Analysis

The obtained lysates were electrophoresed in 10% SDS polyacrylamide gels in an electrophoresis buffer (25 mM Tris-HCl, 143 mM glycine, 0.1% SDS) using the Mini-Protean^®^ Tetra System by Bio-Rad (Hercules, CA, USA) at a constant voltage of 100 V for about 90 min. All reagents were purchased from Sigma-Aldrich (St Louis, MO, USA) unless otherwise stated. In the next step, proteins were transferred (100 V, 100 min) to polyvinylidene fluoride membrane (PVDF, Immobilon^®^-FL Transfer Membranes, Darmstadt, Germany). Membranes were blocked in blocking buffer (5% non-fat dried milk in Tris-buffered saline—0.1% Tween 20 (TBS-T), pH 7.6) for 1 h at room temperature and incubated at 4 °C overnight with primary antibodies for phosphorylated NMDAR1 (rabbit anti-NMDAR1 phospho Ser896; 1:500; cat. no. ab 75680, Abcam, Cambridge, UK) and phosphorylated MOR (rabbit anti-MOR phopsho Ser375; 1:1000; cat. no. 3451, Cell Signaling Technology, Danvers, MA, USA) diluted in blocking buffer. Then, the membranes were washed four times in TBS-T buffer and incubated for 1 h at room temperature with a secondary anti-rabbit antibody (1:3000; cat. no. #7074, Cell Signaling Technology, Danvers, MA, USA) conjugated to horseradish peroxidase. The chemiluminescent detection process was performed using WesternBright™ ECL reagents from Advansta (San Jose, CA, USA). The obtained results were recorded on the Carestream X-ray film (Rochester, NY, USA).

Then, in order to investigate total NMDAR and total MOR, the membranes were stripped using RestoreTM PLUS Western Blot Stripping Buffer from Thermo Scientific (Rockford, IL, USA). The effectiveness of stripping was confirmed at the beginning of the study by developing the stripped membranes. As no bands were detected, the stripping procedure was used over the study. Nevertheless, occasionally the randomly chosen stripped membranes were developed to confirm that the stripping procedure was effective during the whole study. Next, the membranes were reprobed with primary antibodies for NMDAR (1:1000; cat. no. ab 109182, Abcam, Cambridge, UK) and MOR (1:500; cat. no. ab 177898, Abcam, Cambridge, UK). The further procedure was as above.

### 4.8. Enzyme-Linked Immunosorbent Assays (ELISA): Quantification of Protein Kinase A and Protein Kinase C Activity

Changes in the level of PKA and PKC in the STZ-treated rats after administration of tested compounds were assessed using commercially available kits from Abcam, Cambridge, UK (PKA (ab 139435) and PKC (ab 139437)). The assay procedure was strictly following the instructions of the kits.

### 4.9. Data Analysis

All statistical calculations were performed using GraphPad Prism 5 (GraphPad, San Diego, CA, USA). Results from behavioral experiments were expressed as means ± SEM. Statistical analysis was performed by two-way ANOVA followed by the Bonferroni post-hoc test. For Western blot assay, optic density was measured using the Image LabTM Software version 5.2 from Bio-Rad (Hercules, CA, USA). Results were expressed as the ratio of the phosphorylated form to the total form of the tested proteins (pMOR/MOR and pNR1/NR1) related to the STZ-treated rats. Changes in the activity of PKA and PKC were presented as relative values compared to the STZ-treated rats. Statistical analyses were conducted using one-way ANOVA followed by Dunnett’s post-hoc test. Differences were considered significant at * *p* < 0.05, ** *p* < 0.01, and *** *p* < 0.001.

## 5. Conclusions

The processes presented in our study, relating to two opposing receptors, may have a significant effect on designing the bifunctional drugs containing the NMDAR antagonist and the MOR agonist. We suggest that blocking the NMDAR channel by Mg^2+^ restores the MOR-NMDAR complex crucial for MRF analgesia in neuropathic rats. It is worth noting that Mg^2+^ has a high safety profile compared to synthetic NMDAR antagonists. The mechanisms of action described in the paper are a justification for the inclusion of Mg^2+^ into opioid therapy, especially during the treatment of opioid-resistant neuropathic pain.

## Data Availability

Data are contained within the article.

## References

[1] Arnér S., Meyerson B.A. (1988). Lack of analgesic effect of opioids on neuropathic and idiopathic forms of pain. Pain.

[2] Morrone L., Scuteri D., Rombola L., Mizoguchi H., Bagetta G. (2017). Opioids Resistance in Chronic Pain Management. Curr. Neuropharmacol..

[3] Yekkirala A.S., Roberson D.P., Bean B.P., Woolf C.J. (2017). Breaking barriers to novel analgesic drug development. Nat. Rev. Drug Discov..

[4] Dickenson A.H. (1997). NMDA receptor antagonists: Interactions with opioids. Acta Anaesthesiol. Scand..

[5] Mao J. (1999). NMDA and opioid receptors: Their interactions in antinociception, tolerance and neuroplasticity. Brain Res. Rev..

[6] Portenoy R.K., Foley K.M., Inturrisi C.E. (1990). The nature of opioid responsiveness and its implications for neuropathic pain: New hypotheses derived from studies of opioid infusions. Pain.

[7] Lavand’Homme P., Steyaert A. (2017). Opioid-free anesthesia opioid side effects: Tolerance and hyperalgesia. Best Pract. Res. Clin. Anaesthesiol..

[8] Mercadante S., Arcuri E., Santoni A. (2019). Opioid-Induced Tolerance and Hyperalgesia. CNS Drugs.

[9] Nee J., Rangan V., Lembo A. (2018). Reduction in pain: Is it worth the gain? The effect of opioids on the GI tract. Neurogastroenterol. Motil..

[10] Kiyatkin E.A. (2019). Respiratory depression and brain hypoxia induced by opioid drugs: Morphine, oxycodone, heroin, and fentanyl. Neuropharmacology.

[11] Begon S., Pickering G., Eschalier A., Dubray C. (2000). Magnesium and MK-801 have a similar effect in two experimental models of neuropathic pain. Brain Res..

[12] Mao J., Price D., Hayes R.L., Lu J., Mayer D.J., Frenk H. (1993). Intrathecal treatment with dextrorphan or ketamine potently reduces pain-related behaviors in a rat model of peripheral mononeuropathy. Brain Res..

[13] Mizoguchi H., Watanabe C., Yonezawa A., Sakurada S. (2009). New Therapy for Neuropathic Pain. Int. Rev. Neurobiol..

[14] Sang C.N. (2000). NMDA-Receptor Antagonists in Neuropathic Pain: Experimental Methods to Clinical Trials. J. Pain Symptom Manag..

[15] Swartjes M., Morariu A., Niesters M., Aarts L., Dahan A. (2011). Nonselective and NR2B-selective N -methyl-d-aspartic Acid Receptor Antagonists Produce Antinociception and Long-term Relief of Allodynia in Acute and Neuropathic Pain. Anesthesiology.

[16] Martinez V., Christensen D., Kayser V. (2002). The glycine/NMDA receptor antagonist (+)-HA966 enhances the peripheral effect of morphine in neuropathic rats. Pain.

[17] Nichols M.L., Lopez Y., Ossipov M.H., Bian D., Porreca F. (1997). Enhancement of the antiallodynic and antinociceptive efficacy of spinal morphine by antisera to dynorphin A (1–13) or MK-801 in a nerve-ligation model of peripheral neuropathy. Pain.

[18] Yamamoto T., Yaksh T.L. (1992). Studies on the spinal interaction of morphine and the NMDA antagonist MK-801 on the hyperesthesia observed in a rat model of sciatic mononeuropathy. Neurosci. Lett..

[19] Rondón L.J., Privat A.M., Daulhac L., Davin N., Mazur A., Fialip J., Eschalier A., Courteix C. (2010). Magnesium attenuates chronic hypersensitivity and spinal cord NMDA receptor phosphorylation in a rat model of diabetic neuropathic pain. J. Physiol..

[20] Xiao W.-H., Bennett G.J. (1994). Magnesium suppresses neuropathic pain responses in rats via a spinal site of action. Brain Res..

[21] Brill S., Sedgwick P.M., Hamann W., Di Vadi P.P. (2002). Efficacy of intravenous magnesium in neuropathic pain. Br. J. Anaesth..

[22] Yousef A.A., Al-Deeb A.E. (2013). A double-blinded randomised controlled study of the value of sequential intravenous and oral magnesium therapy in patients with chronic low back pain with a neuropathic component. Anaesthesia.

[23] Begon S., Pickering G., Eschalier A., DuBray C. (2002). Magnesium Increases Morphine Analgesic Effect in Different Experimental Models of Pain. Anesthesiology.

[24] Bujalska M., Makulska-Nowak. H., Gumułka S.W. (2009). Magnesium ions and opioid agonists in vincristine-induced neuropathy. Pharmacol. Rep..

[25] Bujalska M., Malinowska E., Makulska-Nowak H., Gumułka S.W. (2008). Magnesium Ions and Opioid Agonist Activity in Streptozotocin-Induced Hyperalgesia. Pharmacology.

[26] Ulugol A., Aslantas A., Ipci Y., Tuncer A., Karadag C.H., Dokmeci I. (2002). Combined systemic administration of morphine and magnesium sulfate attenuates pain-related behavior in mononeuropathic rats. Brain Res..

[27] Williams J.T., Ingram S.L., Henderson G., Chavkin C., Von Zastrow M., Schulz S., Koch T., Evans C.J., Christie M.J. (2013). Regulation of µ-Opioid Receptors: Desensitization, Phosphorylation, Internalization, and Tolerance. Pharmacol. Rev..

[28] Mann A., Illing S., Miess E., Schulz S. (2014). Different mechanisms of homologous and heterologous μ-opioid receptor phosphorylation. Br. J. Pharmacol..

[29] Schulz S., Mayer D., Pfeiffer M., Stumm R., Koch T., Höllt V. (2004). Morphine induces terminal μ-opioid receptor desensitization by sustained phosphorylation of serine-375. EMBO J..

[30] Hansen K.B., Yi F., Perszyk R., Furukawa H., Wollmuth L.P., Gibb A., Traynelis S.F. (2018). Structure, function, and allosteric modulation of NMDA receptors. J. Gen. Physiol..

[31] Paoletti P., Neyton J. (2007). NMDA receptor subunits: Function and pharmacology. Curr. Opin. Pharmacol..

[32] Tingley W.G., Ehlers M.D., Kameyama K., Doherty C., Ptak J.B., Riley C.T., Huganir R.L. (1997). Characterization of Protein Kinase A and Protein Kinase C Phosphorylation of the N-Methyl-D-aspartate Receptor NR1 Subunit Using Phosphorylation Site-specific Antibodies. J. Biol. Chem..

[33] Wang J.Q., Guo M.-L., Jin D.-Z., Xue B., Fibuch E.E., Mao L.-M. (2014). Roles of subunit phosphorylation in regulating glutamate receptor function. Eur. J. Pharmacol..

[34] Lan J.-Y., Skeberdis V.A., Jover T., Grooms S.Y., Lin Y., Araneda R., Zheng X., Bennett M.V.L., Zukin R.S. (2001). Protein kinase C modulates NMDA receptor trafficking and gating. Nat. Neurosci..

[35] Zou X., Lin Q., Willis W.D. (2004). Effect of protein kinase C blockade on phosphorylation of NR1 in dorsal horn and spinothalamic tract cells caused by intradermal capsaicin injection in rats. Brain Res..

[36] Kow L.-M., Commons K., Ogawa S., Pfaff D. (2002). Potentiation of the excitatory action of NMDA in ventrolateral periaqueductal gray by the μ-opioid receptor agonist, DAMGO. Brain Res..

[37] Koyama S., Akaike N. (2008). Activation of μ-opioid receptor selectively potentiates NMDA-induced outward currents in rat locus coeruleus neurons. Neurosci. Res..

[38] Martin G., Nie Z., Siggins G.R. (1997). μ-opioid Receptors Modulate NMDA Receptor-Mediated Responses in Nucleus Accumbens Neurons. J. Neurosci..

[39] Rodríguez-Muñoz M., Sánchez-Blázquez P., Vicente-Sánchez A., Berrocoso E., Garzón J. (2011). The μ-Opioid Receptor and the NMDA Receptor Associate in PAG Neurons: Implications in Pain Control. Neuropsychopharmacology.

[40] Sánchez-Blázquez P., Rodríguez-Muñoz M., Berrocoso E., Garzón J. (2013). The plasticity of the association between μ-opioid receptor and glutamate ionotropic receptor N in opioid analgesic tolerance and neuropathic pain. Eur. J. Pharmacol..

[41] Sánchez-Blázquez P., Rodríguez-Muñoz M., Garzón J. (2010). μ-Opioid Receptors Transiently Activate the Akt-nNOS Pathway to Produce Sustained Potentiation of PKC-Mediated NMDAR-CaMKII Signaling. PLoS ONE.

[42] Rodríguez-Muñoz M., de la Torre-Madrid E., Sánchez-Blázquez P., Garzón J. (2011). NO-released Zinc Supports the Simultaneous Binding of Raf-1 and PKCγ Cysteine-Rich Domains to HINT1 Protein at the μ-Opioid Receptor. Antioxid. Redox Signal..

[43] Rodríguez-Muñoz M., De La Torre-Madrid E., Sánchez-Blázquez P., Wang J.B., Garzón J. (2008). NMDAR-nNOS generated zinc recruits PKCgamma to the HINT1–RGS17 complex bound to the C terminus of μ-opioid receptors. Cell. Signal..

[44] Sánchez-Blázquez P., Rodríguez-Muñoz M., Montero C., De La Torre-Madrid E., Garzón J. (2008). Calcium/calmodulin-dependent protein kinase II supports morphine antinociceptive tolerance by phosphorylation of glycosylated phosducin-like protein. Neuropharmacology.

[45] Just S., Illing S., Trester-Zedlitz M., Lau E.K., Kotowski S.J., Miess E., Mann A., Doll C., Trinidad J.C., Burlingame A.L. (2012). Differentiation of Opioid Drug Effects by Hierarchical Multi-Site Phosphorylation. Mol. Pharmacol..

[46] Garzón J., Rodríguez-Muñoz M., Sánchez-Blázquez P. (2008). Do pharmacological approaches that prevent opioid tolerance target different elements in the same regulatory machinery?. Curr. Drug Abus. Rev..

[47] Yang J., A Bauer B., Wahner-Roedler D.L., Chon T.Y., Xiao L. (2020). The Modified WHO Analgesic Ladder: Is It Appropriate for Chronic Non-Cancer Pain?. J. Pain Res..

[48] Bujalska-Zadrożny M., Tatarkiewicz J., Kulik K., Filip M., Naruszewicz M. (2017). Magnesium enhances opioid-induced analgesia —What we have learnt in the past decades?. Eur. J. Pharm. Sci..

[49] Lueptow L., Fakira A., Bobeck E.N. (2018). The Contribution of the Descending Pain Modulatory Pathway in Opioid Tolerance. Front. Neurosci..

[50] Lee K.-S., Huang Y.-H., Yen C.-T. (2012). Periaqueductal gray stimulation suppresses spontaneous pain behavior in rats. Neurosci. Lett..

[51] Bujalska-Zadrożny M., Duda K. (2014). Additive Effect of Combined Application of Magnesium and MK-801 on Analgesic Action of Morphine. Pharmacology.

[52] Parsons C.G. (2001). NMDA receptors as targets for drug action in neuropathic pain. Eur. J. Pharmacol..

[53] Christrup L. (1997). Morphine metabolites. Acta Anaesthesiol. Scand..

[54] Lötsch J., Geisslinger G. (2001). Morphine-6-Glucuronide: An analgesic of the future?. Clin. Pharmacokinet..

[55] Cheng P.Y., Liu-Chen L.-Y., Pickel V.M. (1997). Dual ultrastructural immunocytochemical labeling of mu and delta opioid receptors in the superficial layers of the rat cervical spinal cord. Brain Res..

[56] Garzón J., Rodríguez-Muñoz M., Sánchez-Blázquez P. (2005). Morphine alters the selective association between μ-opioid receptors and specific RGS proteins in mouse periaqueductal gray matter. Neuropharmacol..

[57] Gomes I., Jordan B.A., Gupta A., Trapaidze N., Nagy V., Devi L.A. (2000). Heterodimerization of mu and delta Opioid Receptors: A Role in Opiate Synergy. J. Neurosci..

[58] Traynor J., Elliott J. (1993). δ-Opioid receptor subtypes and cross-talk with μ-receptors. Trends Pharmacol. Sci..

[59] Fundytus M.E., Schiller P.W., Shapiro M., Weltrowska G., Coderre T.J. (1995). Attenuation of morphine tolerance and dependence with the highly selective δ-opioid receptor antagonist TIPP[ψ]. Eur. J. Pharmacol..

[60] Gucker S., Bidlack J.M. (1992). Protein kinase C activation increases the rate and magnitude of agonist-induced δ-opioid receptor down-regulation in NG108-15 cells. Mol. Pharmacol..

[61] Gao X., Kim H.K., Chung J.M., Chung K. (2005). Enhancement of NMDA receptor phosphorylation of the spinal dorsal horn and nucleus gracilis neurons in neuropathic rats. Pain.

[62] Gao X., Kim H.K., Chung J.M., Chung K. (2007). Reactive oxygen species (ROS) are involved in enhancement of NMDA-receptor phosphorylation in animal models of pain. Pain.

[63] Scott D.B., Blanpied T., Ehlers M.D. (2003). Coordinated PKA and PKC phosphorylation suppresses RXR-mediated ER retention and regulates the surface delivery of NMDA receptors. Neuropharmacology.

[64] De Gregori S., De Gregori M., Ranzani G., Allegri M., Minella C., Regazzi M. (2011). Morphine metabolism, transport and brain disposition. Metab. Brain Dis..

[65] Oppelt W.W., MacIntyre I., Rall D.P. (1963). Magnesium exchange between blood and cerebrospinal fluid. Am. J. Physiol..

[66] Nakhoda A., Wong H.A. (1979). The induction of diabetes in rats by intramuscular administration of streptozotocin. Experientia.

[67] Randall L., Selitto J.J. (1957). A method for measurement of analgesic activity on inflamed tissue. Arch. Int. Pharmacodyn. Ther..

[68] Rodríguez-Muñoz M., de la Torre-Madrid E., Sánchez-Blázquez P., Garzón J. (2007). Morphine Induces Endocytosis of Neuronal μ-opioid Receptors Through the Sustained Transfer of Gα Subunits to RGSZ2 Proteins. Mol. Pain.

[69] Sánchez-Blázquez P., Rodríguez-Díaz M., López-Fando A., Rodriguez-Muñoz M., Garzón J. (2003). The GBeta5 subunit that associates with the R7 subfamily of RGS proteins regulates μ-opioid effects. Neuropharmacology.

